# Social considerations in surgical management of Flood syndrome: a case report

**DOI:** 10.1093/jscr/rjad216

**Published:** 2023-04-24

**Authors:** Setareh Ekhteraei, Ahmed Alsafar

**Affiliations:** School of Medicine, University of Colorado, Denver 80045, CO, USA; School of Medicine, University of Colorado, Denver 80045, CO, USA

## Abstract

Flood syndrome, first described by Dr. Frank Flood in 1961, is a rare condition involving the leakage of ascitic fluid through a ruptured ventral hernia. Most commonly, it occurs in patients with advanced, decompensated liver cirrhosis leading to significant amounts of ascites. Currently, there is no standard of care for Flood syndrome due to its very rare nature. Our case report details the medical, surgical and social aspects of a 45-year-old unhoused male with Flood syndrome with post-surgical complications and subsequent infection. This paper aims to add to the sparse literature on Flood syndrome and to discuss some of the complications and treatment approaches for this condition.

## INTRODUCTION

Liver cirrhosis is a process in which chronic fibrosis of normal liver tissue occurs, resulting in shunting of blood flow from arterial and portal systems to hepatic outflow veins. Common and potentially life-threatening complications of cirrhosis due to portal hypertension include build-up of ascites, spontaneous bacterial peritonitis, esophageal varices and hepatic encephalopathy [1]. The incidence of umbilical hernias in cirrhosis is about 20%, which is 10 times higher than the incidence in the general population [2]. In rare cases of decompensated cirrhosis, large volumes of ascites lead to rupture of the hernia and leakage of ascitic fluid through a skin lesion. This complication is known as Flood syndrome.

Given the rarity of this condition, the approach to management is not standardized and the majority of published data stems from case reports and case series [3, 4]. Prior reviews reported that medical-only management had mortality rates of 60–80% compared with 6–20% involving combined medical and surgical intervention [2].

Conservative medical management may include salt restriction, diuretics, electrolyte monitoring, wound dressings, antibiotics, ostomy pouches and fibrin glue. Multiple surgical approaches have been attempted including pigtail drains, partial splenic embolization [5], temporary percutaneous peritoneal drainage, peritoneovenous shunt, transjugular intrahepatic portosystemic shunt and most commonly, repair of the umbilical hernia with or without a mesh [4].

## CASE REPORT

A 45-year-old unhoused male with a past medical history including cirrhosis secondary to severe alcohol use disorder, resolved hepatitis C infection and ventral hernia status-post open repair 6 months prior to initial presentation was found to have Flood syndrome in the setting of severely decompensated cirrhosis.

The patient initially presented with a 1-day history of fever, rigors and peri-umbilical abdominal pain. He was found to have *Escherichia coli* bacteremia of unknown origin and was discharged with an abdominal binder after treatment. One month after the initial presentation, the patient re-presented to the emergency department with a ruptured umbilical hernia and Flood syndrome involving a 1–2 L loss of ascites from the umbilicus. He was taken emergently to the operating room for exploratory laparotomy and umbilical hernia repair. Operatively, the patient was found to have two small umbilical hernia defects separated by a small bridge of tissue (measuring total 3 × 2 cm). After standard hernia repair without mesh and removal of 2.2 L of ascitic fluid, a 19 french blake drain was placed through the abdominal wall and into the abdomen. The drain, which was to offload intra-abdominal pressure and allow healing of the incision, was planned to be set to gravity and emptied every 6 hours with a goal of maximum 2 L daily output. The patient also received intravascular albumin to modulate body compartment fluid distribution. After a few days, the patient experienced wound dehiscence and abrupt leakage of a large amount of ascites that could not be controlled with negative pressure therapy. He was returned to the operating room, and a small amount of hematoma was removed. A 1 mm area of fascial opening and dehiscence was noted, though no sutures had pulled through his fascial layers. A small incision was made on the lower left quadrant (opposite to the initial drain site), and a tonsil was used to make a tract through the subcutaneous tissue into peritoneum. A new 19Fr Blake drain was then placed. Fascial closure involved interrupted 0-polydioxanone, followed by multi-layer closure with interrupted 3-0 vicryl, and skin closure with 4-0 running monocryl and Dermabond.

His course was then complicated by ileus. However, the patient began declining nasogastric tube placement and total parenteral nutrition. Furthermore, his shelter threatened to discard the patient’s belongings despite provider discussion with the shelter. The patient began communicating his desire to leave against medical advice (AMA), and despite provider discussion with the patient, he left AMA with the drain in place. Three days after discharge, the patient re-presented and was found to have sepsis secondary to Methicillin-resistant *Staphylococcus aureus* peritonitis, which was treated successfully with vancomycin and linezolid before eventual drain removal and discharge.

## DISCUSSION

Certain populations with cirrhosis have an inherently higher risk of hernia rupture and subsequent Flood syndrome. Risk factors include the presence of prior hernia or weakened abdominal muscles. Other medical and social comorbidities that increase abdominal pressures, weaken abdominal wall muscles or prevent bedrest, including epilepsy, chronic constipation, malnutrition, homelessness, and occupation, are also risk factors for hernia rupture [2]. Lastly, acute increases in intra-abdominal pressure, due to abdominal trauma or other causes, may also contribute to rupture [2].

Management of Flood syndrome remains predominantly surgical in nature due to the acutely elevated risk of death and lower efficacy of medical management. This case report highlights a 45-year-old unhoused patient with a past history of a ventral hernia and decompensated cirrhosis complicated by Flood syndrome. Following inadequate attempts at prevention of complications via the use of an abdominal binder and lifestyle modifications to reduce ascitic pressure on abdominal musculature, the patient eventually required repeat hernia repair with drain placement.

Despite successful repair of subsequent fascial wound dehiscence and drain replacement, the patient’s decision to leave AMA with the drain still in place severely complicated his course of healing. It may be worthwhile to consider the significantly increased risk of peritonitis in patients with indwelling peritoneal drains in the management of Flood syndrome [6]. Outpatient drain management is not only difficult, but may not be at all feasible in certain settings where patients lack access to the resources to safely care for a peritoneal drain.

## Data Availability

The data that support the findings of this study are available on request from the corresponding author, SE.

## References

[1] Schuppan D , Afdhal NH. Liver cirrhosis. Lancet 2008;371:838–51. 18328931 10.1016/S0140-6736(08)60383-9 PMC2271178

[2] Strainiene S , Peciulyte M, Strainys T, Stundiene I, Savlan I, Liakina V, et al. Management of Flood syndrome: what can we do better? World J Gastroenterol 2021;27:5297–305. 34539133 10.3748/wjg.v27.i32.5297 PMC8409160

[3] Gorji L , Brown J. Flood syndrome: a rare case of spontaneous rupture of umbilical hernia in a cirrhotic patient. J Surg Case Rep 2022;2022:rjac265. 35712608 10.1093/jscr/rjac265 PMC9197296

[4] Lee JL , Jiang J. Flood syndrome. Gastroenterology Res 2022;15:217–24. 36128184 10.14740/gr1508 PMC9451583

[5] Chikamori F , Mizobuchi K, Ueta K, Takasugi H, Yukishige S, Matsuoka H, et al. Flood syndrome managed by partial splenic embolization and percutaneous peritoneal drainage. Radiol Case Rep 2021;16:108–12. 33204382 10.1016/j.radcr.2020.10.045 PMC7649598

[6] Kathpalia P , Bhatia A, Robertazzi S, Ahn J, Cohen SM, Sontag S, et al. Indwelling peritoneal catheters in patients with cirrhosis and refractory ascites. Intern Med J 2015;45:1026–31. 26122531 10.1111/imj.12843

